# Feasibility study of dual-accelerated simultaneous multi-slice imaging in diffusion tensor imaging of glioma

**DOI:** 10.3389/fonc.2024.1331578

**Published:** 2024-09-20

**Authors:** Yakun He, Xiaoyu Chen, Siqing Yi, Min Wang, Jin Ren, Peng Zhou, Heping Deng

**Affiliations:** Sichuan Cancer Hospital and Institute, Sichuan Cancer Center, School of Medicine, University of Electronic Science and Technology of China, Chengdu, Sichuan, China

**Keywords:** magnetic resonance imaging, simultaneous multi-slice, diffusion tensor imaging, glioma, SNR, CNR

## Abstract

**Objective:**

To explore the value of dual-accelerated simultaneous multi-slice (SMS) imaging in diffusion tensor imaging (DTI) of glioma.

**Methods:**

Thirty-four patients with glioma who underwent magnetic resonance imaging (MRI) in our hospital from January 2022 to March 2023 were randomly selected. The results of dual-accelerated SMS-DTI and conventional DTI were retrospectively analyzed. All patients were scanned using a uMR790 3.0T MRI scanner, and the scanning technicians followed a predefined sequence to ensure consistency in scan parameters. The images were subjectively evaluated using a Likert 5-point scoring system. Objective evaluation was performed by measuring the required values of the images with b-value = 1000 s/mm^2^, primarily measuring the signal intensity in the tumor region and the contralateral normal brain white matter region. The standard deviation values were used to calculate the signal-to-noise ratio (SNR) and contrast-to-noise ratio (CNR) in the same encoding direction as the background noise. The number of generated fiber pathways, fractional anisotropy (FA), and mean diffusivity (MD) were measured and analyzed using post-processing software. The relative FA (rFA) and relative MD (rMD) were calculated.

**Results:**

The results of conventional DTI and SMS-accelerated DTI were compared. In terms of subjective evaluation, including overall image quality, tumor edge clarity, and magnetic sensitivity artifacts, both techniques showed no significant differences, indicating comparable diagnostic performance in anatomical visualization. In terms of objective evaluation and quantitative parameter measurement, there were statistically significant differences in SNR and CNR values, with slightly lower values in the dual-accelerated SMS-DTI compared with conventional DTI, a significant reduction in scanning time can be achieved through a slight loss in image quality. The number of fiber pathways and the rFA and rMD values did not show typical differences between the two techniques. The correlation between these measures was highly similar, with no significant differences observed.

**Conclusion:**

The application of dual-accelerated simultaneous multi-slice imaging in DTI of glioma is feasible.

## 1 Introduction

Glioma is currently the most common primary intracranial tumor. According to the 2018 report from the National Health Commission of China, the annual incidence rate of glioma in China is 5-8 per 100,000, and the 5-year mortality rate is second only to pancreatic cancer and lung cancer among all systemic tumors, posing a serious threat to the life and health of the Chinese population (1). Gliomas originate from glial cells and typically exhibit characteristics consistent with infiltration of adjacent white matter fiber bundles. Conventional magnetic resonance imaging (MRI) has limited ability to differentiate between the tumor edema area and the infiltrating area around the tumor, which may lead to underestimation of the malignancy of the tumor (2–5). Diffusion tensor imaging (DTI), an advanced MRI technique based on the principle of anisotropic diffusion of water molecules within axons, provides fractional anisotropy (FA) and mean diffusivity (MD) values, which are commonly used in glioma evaluation. These parameters can change based on the invasiveness and destructiveness of gliomas (6–8). FA and MD values also play an important role in glioma grading (9, 10). DTI can also provide information about the involvement of intracranial nerve fiber tissue and cortical functional areas, which is of great significance for the differential diagnosis, determination of surgical boundaries, prognosis assessment, and monitoring of treatment effects in brain gliomas. It is an important supplement to morphological imaging diagnosis (11, 12). However, DTI requires data with more than six diffusion directions to calculate the fiber bundle trajectory, resulting in longer examination time and higher cooperation requirements for glioma patients. Some studies have reported that simultaneous multi-slice (SMS) acquisition technique, combined with different MRI sequences such as DTI, vascular imaging, and elastography, can significantly improve scan speed (13–16). This study aims to compare the feasibility of dual-acceleration SMS-DTI with conventional DTI in glioma DTI.

## 2 Materials and methods

### 2.1 General information

Retrospective analysis was conducted on 34 patients diagnosed with brain gliomas from January 2022 to March 2023 in our hospital. The histopathological diagnosis agreed with the radiological diagnosis initially proposed by radiologists. The age of the patients ranged from 27 to 58 years, with an average of (43.6 ± 5.8) years. The pathological grading included low-grade gliomas (LGG, grades I to II) and high-grade gliomas (HGG, grades III to IV) (17). Among them, there were 23 cases (14.3%) of HGG and 11 cases (85.7%) of LGG, The study was carried out in accordance with the principles of the Declaration of Helsinki. This study was approved by the medical ethics committee of Sichuan Cancer Hospital (SCCHEC-02-2024-086).

### 2.2 Methods

All patients underwent MR imaging using a 3.0T MRI scanner (uMR790; United Imaging, Shanghai, China) in the supine position with head first. A 32-channel dedicated head coil was used for whole-brain imaging. Axial scans of conventional T1-weighted imaging (T1WI FLAIR), T2-weighted imaging (T2WI), T2 FLAIR, conventional DTI, and dual-acceleration SMS-DTI were performed. Axial, sagittal, and coronal scans of contrast-enhanced T1WI were also performed. Conventional DTI and dual-acceleration SMS-DTI were scanned before contrast agent injection. The scanning parameters were set according to a fixed sequence package to ensure consistency. See **Table 1** for detailed imaging parameter settings.

**Table 1:** Scanning parameters of conventional DTI and doubled-acceleration SMS-DTI sequences.

	Conventional DTI	Doubled-acceleration SMS-DTI	T1WI FLAIR	T2WI	T2 FLAIR	contrast-enhanced T1WI
slices	36	36	21	21	21	160
TR (msec)	4561	2601	1850	5800	8000	7.1
TE (msec)	75	71.5	5.88	124.6	134.64	3.1
Excitation flip angle	90^0^	90^0^	90^0^	90^0^	90^0^	10^0^
Bandwidth	1750Hz	1750Hz	350Hz	260Hz	220Hz	250Hz
Number of diffusion directions	32	32	NO	NO	NO	NO
b-value (s/mm^-2^)	0/1000	0/1000	NO	NO	NO	NO
Readout FOV (mm)	250	250	230	230	230	230
Phase FOV (mm)	230	230	200	200	200	200
Phase encoding direction	A>P	A>P	R>L	R>L	R>L	R>L
Matrix	128*100	128*100	320*240	352*317	288*245	256*256
Slice thickness (mm)	4	4	5	5	5	
Multi-Slice factor	NO	2	NO	NO	NO	NO
Scan time	5:21min	3:03min	59sec	41sec	1:20min	3:32min

Conventional DTI and Doubled-acceleration SMS-DTI are the spin-echo.

### 2.3 Evaluation indicators

#### 2.3.1 Subjective evaluation indicators

Two radiologists with over 10 years of experience in brain tumor diagnosis used the Likert 5-point scoring method to evaluate the overall image quality, tumor edge clarity, and magnetic susceptibility artifacts, without knowing the information of the image sequence (17). The specific scoring criteria are as follows: for overall image quality: 0 for non-diagnostic, 1 for poor, 2 for fair, 3 for good, and 4 for excellent. For tumor edge clarity: 0 for indistinguishable, 1 for unclear anatomical boundaries and difficult to identify, 2 for recognizable but blurry boundaries, 3 for relatively good boundaries, and 4 for clear tumor edges. For magnetic susceptibility artifacts: 0 for severe artifacts significantly affecting diagnostic evaluation, 1 for moderate artifacts with significant impact on diagnostic evaluation, 2 for moderate artifacts with mild impact on diagnosis, 3 for minimal artifacts with no impact on diagnostic evaluation, and 4 for few or no artifacts, **Table 2**.

**Table 2:** Scoring criteria for subjective evaluation.

Scores	overall image quality	tumor edge clarity	magnetic susceptibility artifacts
0	non-diagnostic	indistinguishable	severe artifacts significantly affecting diagnostic evaluation
1	poor	unclear anatomical boundaries and difficult to identify	moderate artifacts with significant impact on diagnostic evaluation
2	fair	recognizable but blurry boundaries	moderate artifacts with mild impact on diagnosis
3	good	relatively good boundaries	minimal artifacts with no impact on diagnostic evaluation
4	excellent	clear tumor edges	few or no artifacts

#### 2.3.2 Objective evaluation indicators

Likewise, two radiologists with over 10 years of experience in brain tumor diagnosis reviewed the images after concealing patient information and conducted measurements on the relevant images of the two types of examinations with b-value = 1000s/mm^2^. They mainly measured the signal intensity of the tumor area and the contralateral normal brain white matter area, and selected the standard deviation value at the same encoding direction as the position of the background noise. The ROI sizes were 0.26-1.04 cm^2^ for the tumor area and 0.26-1.24 cm^2^ for the normal brain white matter signal intensity area, with standard deviation values ranging from 0.97-1.14 cm^2^. During the specific measurement process, three ROIs were selected, avoiding areas of necrosis, hemorrhage, and cystic changes. The average values were then calculated, and SNR (signal-to-noise ratio) and CNR (contrast-to-noise ratio) were calculated using the following formulas:


$$SNR=\frac{SI_{\text{tumor area}}}{SD_{\text{background}}\;noise}$$

$$CNR=\frac{SI_{\text{tumor area}}-SI_{\text{normal brain white matter}}}{SD_{\text{background}}\;noise}$$

#### 2.3.3 Measurement of quantitative parameters

**Figure 1:**
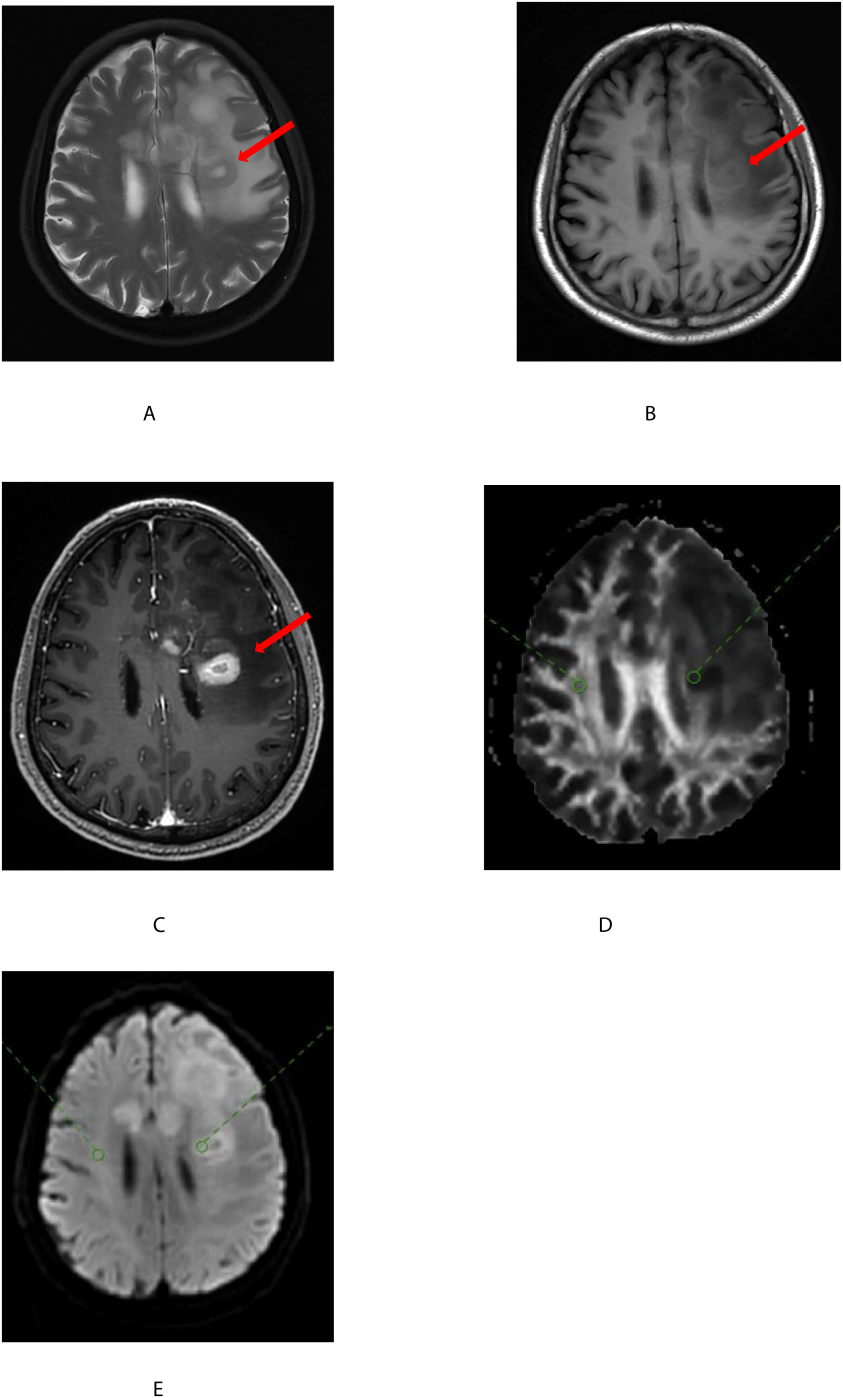
A 68-year-old man with a glioblastoma of the left basal ganglia(shown by the red arrow). T2-weighted images **(A)** on plain scan, T1-weighted images **(B)** on plain scan, and T1-weighted images **(C)** on enhanced scan showed uneven tumor signals with central necrotic foci. On FA **(D)** and MD **(E)**, two regions of interest (ROI) were placed within the solid portion of the tumor(shown by the red circle) and normal white matter(shown by the green circle) and combined with enhanced T1-weighted images were registered.

Likewise, the radiologists with over 10 years of experience in brain tumor diagnosis used post-processing software to measure and analyze the number of fiber bundles, FA (fractional anisotropy) values, and MD (mean diffusivity) values generated in (1). The FA and MD maps were co-registered with the pre- and post-enhanced T1-weighted images and FLAIR images for accurate delineation of regions of interest (ROI). In the FA map with a b-value of 1000s/mm^2^, two ROIs of 20-30 mm^2^ were drawn within the tumor area and normal brain white matter. The tumor area region was defined as the most enhanced area on the enhanced T1-weighted image, avoiding cystic and necrotic areas. In non-enhanced tumors, the ROI was placed within the solid portion. The ROI in the normal brain white matter was placed at the same level as the tumor. The ROIs on the FA map were synchronized with the MD map. The absolute values of FA and MD in the tumor area and normal brain white matter, as well as the relative FA and MD (rFA and rMD), were calculated. The rFA and rMD values were calculated as the ratio of the absolute FA and MD values in the tumor area to those in the normal brain white matter, **Figure 1**.

### 2.4 Statistical methods

All data were processed using the SPSS 26.0 statistical software (IBM, Armonk, New York, USA). Continuous data were expressed as mean ± standard deviation ( $\bar{x}\pm s$ ), and categorical data were expressed as percentages. The intraclass correlation coefficient (ICC) was used to analyze the consistency of subjective and objective evaluation. The value of the ICC was used for evaluation, where a value between 0.82 and 1.00 indicated excellent consistency, 0.61-0.80 indicated good consistency, 0.41-0.60 indicated moderate consistency, 0.21-0.40 indicated poor consistency, and ICC < 0.20 indicated inconsistency. Paired sample T-tests were performed on measurement data lines conforming to orthographic distribution. The Mann-Whitney U test were performed on measurement data that does not conform to positive distribution, with *P*<0.05 indicating statistical significance.

## 3 Results

### 3.1 Consistency analysis of subjective evaluation of the two results

During the review process, it was found that both types of examination results had high ICC values in terms of overall image quality, tumor edge clarity, and susceptibility artifacts, indicating good subjective consistency, **Table 3**.

**Table 3:** Comparison of subjective consistency of the two examination results.

Examination Result	Evaluation Index	Intra-Class Correlation Coefficient (ICC)	95%CI(LL)	95%CI(UL)
DTI	Overall Image Quality	0.827	0.682	0.909
	Tumor Edge Clarity	0.863	0.744	0.929
	Susceptibility Artifacts	0.855	0.711	0.927
SMS-DTI	Overall Image Quality	0.870	0.758	0.933
	Tumor Edge Clarity	0.858	0.734	0.927
	Susceptibility Artifacts	0.846	0.705	0.921

### 3.2 Consistency analysis of objective evaluation of the two results

In terms of objective consistency, the ICC values of the measured data obtained from both types of examination results were significantly greater than 0.7, indicating good objective consistency, **Table 4**.

**Table 4:** Comparison of objective consistency of the two examination results.

Examination Result	Evaluation Index	Intra-Class Correlation Coefficient (ICC)	95%CI(LL)	95%CI(UL)
DTI	SI tumor area	0.855	0.632	0.936
	SI normal brain white matter	0.867	0.671	0.940
	SD background	0.834	0.673	0.916
SMS-DTI	SI tumor area	0.872	0.759	0.934
	SI normal brain white matter	0894	0.737	0.952
	SD background	0.881	0.722	0.945

### 3.3 Comparison of subjective and objective scores of clinical diagnosis between the two examination results

The subjective scoring comparison of the two examination results revealed no significant differences in overall image quality, tumor edge clarity, and susceptibility artifacts, and there was no statistical significance (*P*>0.05). However, there were differences in SNR and CNR values between the two examination results (P<0.05), **Table 5**.

**Table 5:** Comparison and analysis of subjective and objective scores of clinical diagnosis of the two examination results.

Evaluation Index	DTI	SMS-DTI	*t*	*P*
Overall Image Quality	3.00 ± 0.65	3.00 ± 0.68	-0.882	0.378
Tumor Edge Clarity	3.00 ± 0.76	3.00 ± 0.77	0.081	0.935
Susceptibility Artifacts	3.00 ± 0.59	3.00 ± 0.68	0.353	0.724
SNR	9.39 (6.04,13.63)	7.36 (5.06,12.82)	-1.992	0.046
CNR	2.85 (1.34,5.15)	1.06 (0.51,2.81)	-3.274	0.001

### 3.4 Comparison of quantitative parameters

The comparison of fiber counts, rFA, and rMD values obtained from the two examinations in different pathological grades showed no statistically significant differences (*P*>0.05) between double-accelerated SMS-DTI and conventional DTI in HGG and LGG, **Table 6**.

**Table 6:** Comparison of quantitative parameters in pathological grades of the two examination results.

Parameter	HGG			LGG		
	Fiber Count	rFA	rMD	Fiber Count	rFA	rMD
DTI	17330.78 ± 1835.71	0.65 ± 0.27	1.14 ± 0.27	15987.64 ± 3190.01	0.57 ± 0.05	1.12 ± 0.05
SMS-DTI	18201.39 ± 2497.83	0.65 ± 0.22	1.03 ± 0.19	17452.36 ± 1212.75	0.53 ± 0.081.368	1.21 ± 0.2
*t*	1.347	0.072	1.568	1.423	0.187	1.329
*P physicians*	0.185	0.943	0.124	0.170	0.57 ± 0.05	0.199

## 4 Discussion

Due to its ability to display white matter fiber bundles and quantitatively analyze diffusion data, DTI has been recommended for use in patients with brain gliomas, especially in patients with tumors invading brain functional areas, as it provides data that can increase the resection range of the tumor while protecting the patient’s neurological function (18–20). However, DTI requires data with more than six diffusion directions to calculate the fiber bundle trajectory, and the more directions, the more accurate the calculated data, which also leads to longer scanning time. Due to the poor condition of glioma patients, prolonged magnetic resonance examination is also a challenge for patients, so shortening the examination time has become a necessary means for us to perform this MRI functional examination. SMS, also known as multi-bandwidth technology, uses multi-frequency excitation and multiple-channel coil sensitivity information to reconstruct data from different levels, thereby reducing the repetition time (TR) and shortening the examination time from data excitation to collection.

In this study, double-acceleration SMS was applied to DTI. According to subjective evaluation, there was no significant difference between double-acceleration SMS and conventional DTI in overall image quality, tumor edge clarity, and magnetic sensitivity artifacts *(P*<0.05). Physicians evaluated that the image deformation caused by magnetic sensitivity artifacts was heavier in double-acceleration SMS DTI, but the difference was not statistically significant within an acceptable range. The final evaluation images met the anatomical diagnostic criteria. Therefore, the study shows that there is not much difference in subjective evaluation when using accelerated techniques, but the speed has improved significantly.

Objective indicators show that there is a statistically significant difference in signal-to-noise ratio and contrast-to-noise ratio between double-acceleration SMS-DTI and conventional DTI (P>0.05), and they have decreased. Signal-to-noise ratio depends on many factors, including spatial resolution and parameters of specific sequences, such as TR and echo time (TE). Signal-to-noise ratio is significantly positively correlated with voxel size and TR, and significantly negatively correlated with TE. Therefore, reducing TR can reduce the signal-to-noise ratio. We believe that due to the adjustment of TR according to the acceleration factor, which decreases with the increase of the acceleration factor, considering that different sequences need to have the same spatial resolution, bandwidth, etc. to ensure the comparability of parameters between sequences, increasing the acceleration factor will reduce the measured signal-to-noise ratio, which is consistent with the current results. In addition, the short distance between SMS excitation planes may hinder their signal separation, thereby affecting the signal-to-noise ratio. Some scholars have included a correction factor for acquisition time in the calculation of signal-to-noise ratio and contrast-to-noise ratio. Their research shows that after correcting the acquisition time, double-acceleration SMS-DTI has a significantly higher signal-to-noise ratio compared to conventional DTI (21).

The FA and MD values differ between the subcortical and deep white matter, as well as between white matter and gray matter (22, 23). Gliomas may be located in different areas of brain tissue, which may lead to inaccuracies when evaluating FA and MD values. Therefore, we calculated the correlated indices of FA and MD by comparing the tumor solid part with normal brain white matter to limit this inaccuracy. The results showed that there were no typical differences in rFA and rMD values between the two examination results (P>0.05), and their correlation was highly similar without significant differences. Detailed analysis of the results showed that the double-acceleration simultaneous multi-plane acquisition technique effectively reduced the acquisition time, while ensuring image quality and quantitative parameters.

This study has limitations: (1) the sample size is small, and further analysis with a larger sample size is needed in the later stage to obtain more abundant data support. (2) Only a comparison analysis of double-acceleration SMS applied to DTI imaging was performed, and in the future, more acceleration factors should be used for SMS combined with DTI imaging for multi-parameter analysis to determine the optimal scan time for clinical practice, ensuring image quality and reducing scan time simultaneously.

In summary, this study confirms the feasibility of double-acceleration simultaneous multi-plane acquisition technique for DTI in brain gliomas. Its signal-to-noise ratio, quantitative diffusion values, and overall image quality are similar to conventional DTI, but the acquisition speed is faster, which can be widely applied to magnetic resonance diffusion tensor imaging in clinical glioma patients.

## Data Availability

The original contributions presented in the study are included in the article/supplementary material. Further inquiries can be directed to the corresponding author.
